# Feature Genes in Neuroblastoma Distinguishing High-Risk and Non-high-Risk Neuroblastoma Patients: Development and Validation Combining Random Forest With Artificial Neural Network

**DOI:** 10.3389/fmed.2022.882348

**Published:** 2022-07-15

**Authors:** Sha Yang, Lingfeng Zeng, Xin Jin, Huapeng Lin, Jianning Song

**Affiliations:** ^1^Department of Surgery, Children’s Hospital of Chongqing Medical University, Chongqing, China; ^2^Ministry of Education Key Laboratory of Child Development and Disorders, Chongqing, China; ^3^National Clinical Research Center for Child Health and Disorders, Chongqing, China; ^4^China International Science and Technology Cooperation Base of Child Development and Critical Disorders, Chongqing, China; ^5^Chongqing Key Laboratory of Pediatrics, Chongqing, China; ^6^Chongqing Engineering Research Center of Stem Cell Therapy, Chongqing, China; ^7^Children’s Hospital of Chongqing Medical University, Chongqing, China; ^8^Department of Nephrology, The Second Xiangya Hospital of Central South University, Changsha, China; ^9^Department of Cardiacthoracic, Children’s Hospital of Chongqing Medical University, Chongqing, China; ^10^Department of Intensive Care Unit, Affiliated Hangzhou First People’s Hospital, Zhejiang University School of Medicine, Hangzhou, China; ^11^Department of General Surgery, Guiqian International General Hospital, Guiyang, China

**Keywords:** neuroblastoma, random forest, artificial neural network, high-risk category, genes

## Abstract

There is a significant difference in prognosis among different risk groups. Therefore, it is of great significance to correctly identify the risk grouping of children. Using the genomic data of neuroblastoma samples in public databases, we used GSE49710 as the training set data to calculate the feature genes of the high-risk group and non-high-risk group samples based on the random forest (RF) algorithm and artificial neural network (ANN) algorithm. The screening results of RF showed that EPS8L1, PLCD4, CHD5, NTRK1, and SLC22A4 were the feature differentially expressed genes (DEGs) of high-risk neuroblastoma. The prediction model based on gene expression data in this study showed high overall accuracy and precision in both the training set and the test set (AUC = 0.998 in GSE49710 and AUC = 0.858 in GSE73517). Kaplan–Meier plotter showed that the overall survival and progression-free survival of patients in the low-risk subgroup were significantly better than those in the high-risk subgroup [HR: 3.86 (95% CI: 2.44–6.10) and HR: 3.03 (95% CI: 2.03–4.52), respectively]. Our ANN-based model has better classification performance than the SVM-based model and XGboost-based model. Nevertheless, more convincing data sets and machine learning algorithms will be needed to build diagnostic models for individual organization types in the future.

## Introduction

Neuroblastoma (NB) is an embryonal tumor derived from immature embryonic cells of paravertebral sympathetic ganglia or adrenal medulla, accounting for 15% of all childhood cancer deaths (1, 2). The biological heterogeneity of NB is very obvious, and some cases can regress spontaneously, but most of the tumors show occult onset and progress rapidly (3). The International Neuroblastoma Staging System (INSS) divides patients into low, intermediate, and high-risk groups based on prognostic factors (4). There is a significant difference in prognosis among different risk groups (5). The overall survival rate of patients in the low-moderate risk group could be greater than 95% by surgery alone (6), while high-risk children have a poor prognosis, the long-term disease-free survival rate is less than 50%, and the risk of later metastasis and recurrence is higher (7). Therefore, it is of great significance to correctly identify the risk grouping of children. The development and use of the INSS guideline have provided consistency in the staging of NB patients around the world, but the guideline staging is postoperative, and the level of surgery can affect the staging grade of the tumor.

With the rapid development of bioinformatics technology, we have a deeper understanding of neuroblastoma. A large amount of biological data has exploded, various biological databases have been established, and various prediction models can be established using mathematical knowledge (8–11). But there are thousands of genetic data, and screening out the signature genes will help us more quickly and easily distinguish between high-risk and non-high-risk neuroblastoma patients. In order to improve the accuracy and efficiency of tumor pathological diagnosis, Marya et al. (12) proposed an artificial intelligence diagnostic model to identify benign and malignant tumors. Experimental studies found that the diagnostic model had an accuracy of 90% in identifying benign and malignant tumors.

Both random forest (RF) (13) and artificial neural network (ANN) (14) algorithms belong to machine learning. RF algorithm can filter eigengenes and calculate the importance of each eigengene to classification and is suitable for processing large amounts of data (15). RF algorithm is an ensemble machine learning algorithm and an extended variant of bagging (16). First, use the random resampling method Bootstrap and node random splitting method to generate multiple decision trees, and on the basis of building Bagging ensemble with decision tree as the base learner, further introduce random attribute selection in the training process of decision tree, and then adopt the classification results are obtained by voting. Moreover, RF has the ability to analyze complex interaction classification features, has good robustness to data with missing values, and has a very fast learning speed. Its feature importance measure can be used to perform feature selection on high-dimensional data, which has been widely used in various data classifications (17). ANN is a non-linear function model that imitates the behavioral characteristics of biological neural networks and has strong self-learning and adaptive capabilities. There is also a layer of hidden neurons between the input and the output in ANN. Each input node is assigned a weight, and then the sum of the weighted values is added to calculate the output amount for discrimination (18).

Based on the genomic data of neuroblastoma samples in public databases, we used the training set data to calculate the differential genes of the high-risk group and non-high-risk group samples, performed biological function analysis, and assessed the differences in the tumor microenvironment of the two groups of patients, and subsequently, we used RF to find the feature genes of high-risk group in the DEGs between high and non-high risk neuroblastoma, and then used ANN to build a disease prediction model, and then used the test set to verify the accuracy of the model. In addition, we also validated the prognosis of the groupings according to our model, including overall survival (OS) and progression-free survival (PFS), with a dataset with survival data.

## Materials and Methods

### Datasets

The gene profiles of GSE49710 (19) and GSE73517 (20) [GPL16876, Agilent-020382 Human Custom Microarray 44k (Feature Number version)] were obtained from Gene Expression Omnibus (GEO^1^), which is an open functional genomics database. We set GSE49710 as the training cohort, including 176 primary neuroblastomas samples with high-risk category and 322 primary neuroblastomas samples with low-risk category, and we set GSE73517 as the test cohort, including 56 primary neuroblastomas samples with high-risk category and 49 primary neuroblastomas samples with low-risk category. The gene profiles of GSE85047 (21) (GPL5175 Affymetrix Human Exon 1.0 ST Array [transcript (gene) version]) with survival data were also obtained as validation data to validate the prognosis of the groupings according to our model. GSE85047 included 283 primary neuroblastoma samples, of which 276 had overall survival data and 275 had progression-free survival data.

### Identification of Differentially Expressed Genes

After processed and standardized raw data, DEGs between low-risk category and high-risk category primary neuroblastomas samples in the training cohort were identified by “limma” R package (22). The threshold for significant DEGs was as follows: |log2 fold change (FC)| > 2 and adjusted *p*-value < 0.05. A volcano plot and a heatmap were drawn to visualize the analysis results.

### Functional Enrichment Analysis

The “clusterProfiler” R package (23) was applied to carry out Gene Ontology (GO) which included biological process (BP), cellular component (CC), molecular function (MF), and Kyoto Encyclopedia of Genes and Genomes (KEGG) pathway analysis for DEGs. Besides, we used metascape.org^2^ to perform GO and KEGG analyses for DEGs again. A *p* < 0.05 was considered as the threshold. The bar plot was generated to visualize the enrichment analysis results and a Network Diagram was generated to visualize the relationship between enriched terms.

### Construction of Protein–Protein Interaction Network

STRING database^3^ (24) was used to construct the Protein–Protein Interaction (PPI) network to analyze the functional interactions of DEGs. An interaction score > 0.9 was set as significant differences and isolated nodes were removed.

### Comparison of 22 Tumor Immune Cell Subtypes Between Low- and High-Risk Category Groups

“CIBERSORT” R package (25) was used to determine the proportions of 22 tumor immune cell (TIC) subtypes of each sample, and we set the perm at 1000. A *p*-value < 0.05 was considered a significant result. A violin plot and a bar plot were drawn to show the differences in relative expression of 22 TICs between low- and high-risk category groups. The correlation between TICs in TME of primary neuroblastomas was visualized by “corrplot” R package.

### Feature Genes Screened by Random Forest

A balanced iterative random forest algorithm was constructed by “randomForest” R package (26) to select the feature genes from DEGs for the high-risk category of primary neuroblastomas. For the first step, we calculated the average model miscalculation rate of all DEGs. Six nodes was selected as the best variable number for the binary tree, and the best number of trees contained in the random forest was set at 500. For the second step, we used the decreasing accuracy method, also called the Gini coefficient method, to construct a random forest model and obtain the dimensional importance value from the model. The DEGs with an importance value > 2 were chosen as the high-risk category of primary neuroblastomas feature genes for the subsequent analysis. A heatmap was drawn to show the result of the unsupervised hierarchical clusters of the feature genes in the training cohort using “pheatmap” R package. Subsequently, we converted the expression data of the feature genes into a score table called Gene Score. the expression value of feature genes will be converted to 1, when the expression value of an upregulated/downregulated gene in a certain sample is higher/lower than the median expression value of the gene in all samples, otherwise 0.

### Construction of Artificial Neural Network Model

We used “neuralnet” R package (27) to construct an artificial neural network model of the feature genes (important variables), which was composed of one input layer, one hidden layer, and one output layer, to be used in classification and prediction of the high-risk category of primary neuroblastomas. Five hidden nodes were set and rectified linear unit was exploited as an activation function in the hidden layer. And two nodes (Low-/high-risk category of primary neuroblastoma) were set and a softmax function was the activation function of each node in the output layer. In this ANN model, the high-risk category classification score was represented by the sum of the expression levels of the feature genes multiplied by the product of the weight scores. Area under ROC curve (AUC) (28) was calculated using “pROC” R package (29) to assess the discriminative ability of the model. AUC values vary from 0.5 to 1.0, where 0.5 represents random chance and 1.0 indicates a perfect fit. Typically, AUC values greater than 0.70 suggest a reasonable estimation (30).

### Verification of Artificial Neural Network Model

The training cohort (GSE49710) was used to train the ANN model, and the test cohort (GSE73517) was used to test the model. Next, we verified the prognostic effect of the ANN model. According to this model, the validation set (GSE85047) was divided into two groups, which were, respectively, defined as high-risk subgroup and low-risk subgroup. Kaplan–Meier analysis (31) was performed for the high-risk and low-risk subgroups of the validation set using “survimer” R package, and log-rank tests were used to assess statistically significant differences. We evaluated the performance of the ANN model by comparing the predictive results of the extreme gradient boosting (XGBoost) model (32) and Support Vector Machines (SVM) (33) model. All the three classifiers employed in the study are state-of-the-art machine learning techniques that show good performances in various applications. Statistical analyses were conducted using R (version 4.0.5, R Core Team, Vienna, Austria). All machine learning modeling was performed using the Caret package, (34) and the application was built and deployed using the Shiny package and server (35).

## Results

### Identification of Differentially Expressed Genes

**FIGURE 1:**
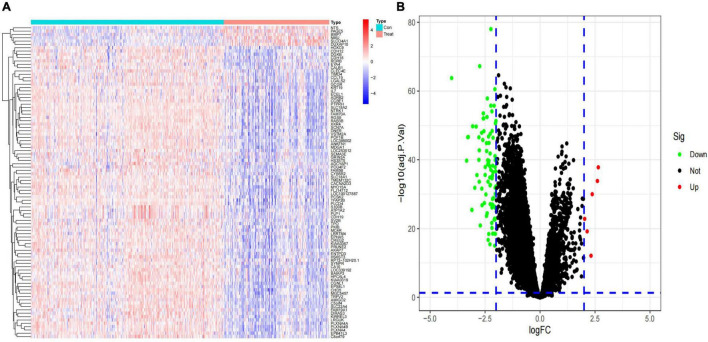
Identification of differentially expressed genes (DEGs) in the training dataset. **(A)** Heat map of the DEGs; **(B)** volcano map of the DEGs.

After standardization of the microarray results from GSE49710, DEGs were identified. (|log2 fold change (FC)| > 2 and adjusted *p*-value < 0.05, Supplementary Table 1) Ultimately, 94 DEGs were detected, most of which (88/94) were downregulated genes in high-risk category primary neuroblastomas samples, while 6 were upregulated genes. The results were validated with a volcano plot of all downregulated genes and upregulated genes (Figure 1A). Figure 1B shows the DEG expression heatmap. The heatmap illustrates the expression profiles of the 94 DEGs in the low-risk and high-risk category groups, with red representing the high-risk category groups, green the low-risk category groups, red the upregulated DEGs, and blue the downregulated DEGs. The volcano plot validated all downregulated genes in the green plot and upregulated genes in the red plot.

### Functional Enrichment Analysis and Protein–Protein Interaction Network Construction

**FIGURE 2:**
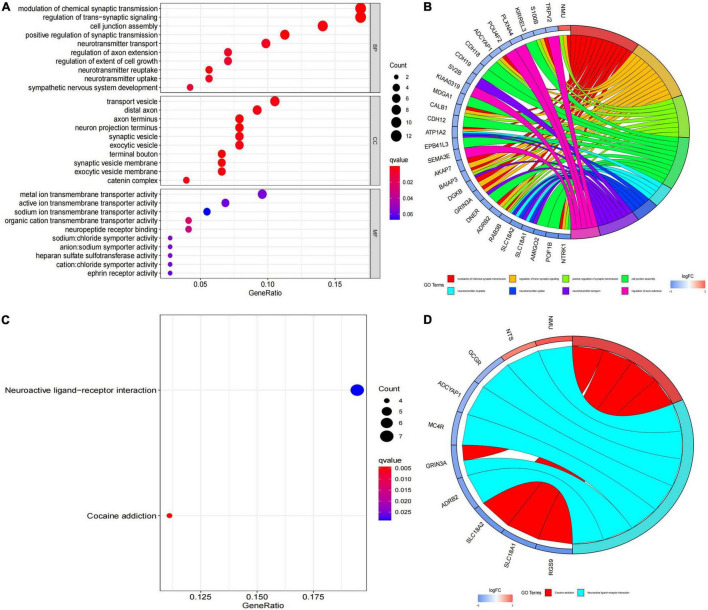
Kyoto Encyclopedia of Genes and Genomes (KEGG) pathway analysis and Gene Ontology (GO) functional enrichment analysis. **(A)** Biological process, cellular component and molecular function annotation diagram of differentially expressed genes (DEGs); **(B)** ring plot showing GO enrichment. The left side indicates the DEGs, the red gene band indicates upregulation, and blue indicates downregulation. The band on the right with different colors represents different GO terms. The connecting line indicates that the gene is included in the GO term; **(C)** KEGG annotation diagram of the DEGs; **(D)** ring plot showing KEGG enrichment. The left side indicates the DEGs, the red gene band indicates upregulation, and blue indicates downregulation. The band on the right with different colors represents different KEGG terms. The connecting line indicates that the gene is included in the KEGG term.

**FIGURE 3:**
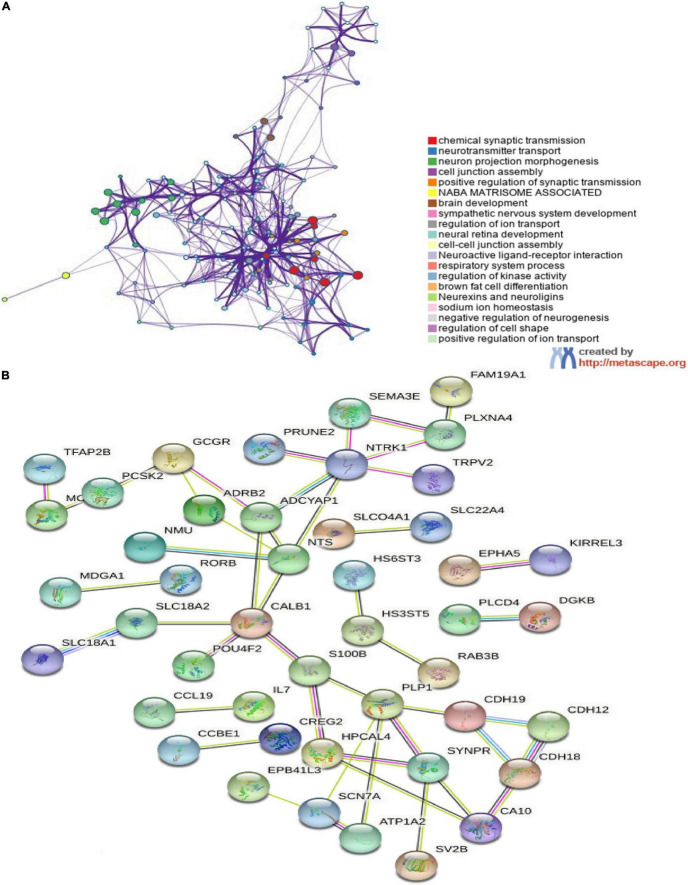
The enrichment analyses in metascape and protein–protein interaction (PPI) analyses in STRING. **(A)** Detailed information relating to changes in the biological function of DEGs in datasets through the enrichment analyses. Network of enriched terms colored by cluster identity, where nodes that share the same cluster identity are typically close to each other; **(B)** PPI network of DEGs. An interaction score > 0.9 was set as significant differences and isolated nodes were removed.

In order to further investigate the biological functions of the 94 DEGs, GO and KEGG analyses were performed. In GO functional enrichment analysis, the DEGs were highly enriched in the modulation of chemical synaptic transmission, regulation of transsynaptic signaling, and neurotransmitter transport/uptake/reuptake (BP); distal axon, vesicle, neuron projection terminus, axon terminus, and terminal bouton (CC); metal ion transmembrane transporter activity, sodium:chloride symporter activity, neuropeptide receptor binding, and organic cation transmembrane transporter activity (MF; Figure 2A). The shared term level and the cluster of the overlap between DEG lists are shown in circos (Figure 2B). In the KEGG enrichment analysis, the DEGs were highly enriched in Neuroactive ligand receptor interaction and Cocaine addiction (Figures 2C,D). Furthermore, the enrichment analysis of the DEGs was performed by metascape, which revealed that these DEGs were markedly enriched in chemical synaptic transmission, neurotransmitter transport, neuron projection morphogenesis, cell junction assembly, Neuroactive ligand-receptor interaction, and regulation of kinase activity (Figure 3A). The PPI network of the DEGs was analyzed by using STRING. The PPI analysis of the DEGs was performed by STRING, which revealed that there were 84 nodes and 46 edges (Figure 3B).

### Immune Cell Infiltration in Primary Neuroblastomas

**FIGURE 4:**
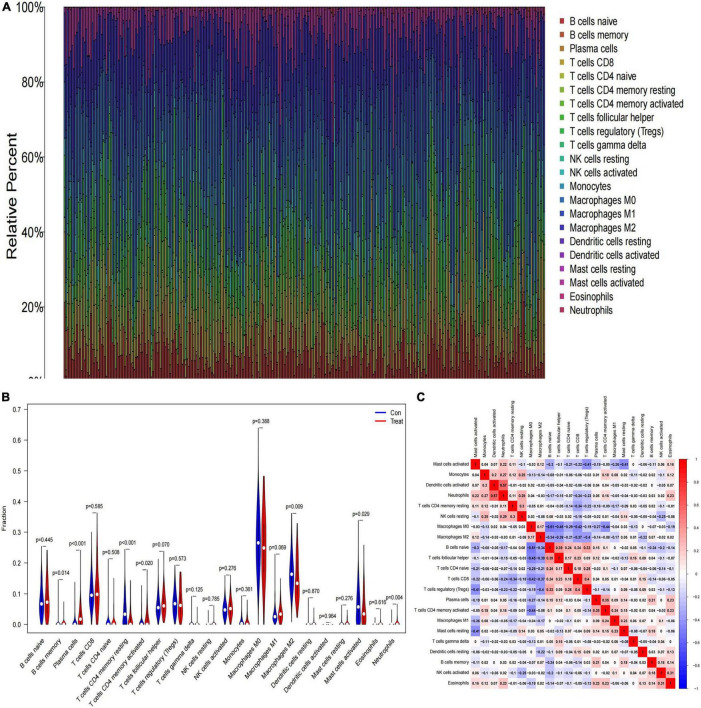
The landscape of immune infiltration between low- and high-risk category groups. **(A)** The relative percentage of 22 subpopulations of immune cells in the training cohort; **(B)** the difference in immune infiltration between low- and high-risk category groups. (The low-risk category group was marked as blue color and the high-risk category group was marked as red color; *p*-values < 0.05 were considered as statistically significant.); **(C)** The correlation of 22 subpopulations of immune cells. Red represents positive correlation; blue represents negative correlation.

We investigated the difference in immune infiltration between high-risk category and low-risk category primary neuroblastomas tissues by using the CIBERSORT algorithm. Figure 4A shows the proportion of 22 subpopulations of immune cells in individual samples, which revealed that there are differences in the infiltration of each sample. Figure 4B shows that compared with low-risk category primary neuroblastomas tissues, a higher proportion of Plasma cells, memory B cells, activated memory CD4 T cells, Neutrophils, and a lower proportion of resting memory CD4 T cells, M2 macrophages, activated mast cells were generally contained in high-risk category primary neuroblastomas tissues. Subsequently, we explored the relationship between each immune cell subtype in the tumor microenvironment (TME; Figure 4C).

### Feature Genes Screened by Random Forest

**FIGURE 5:**
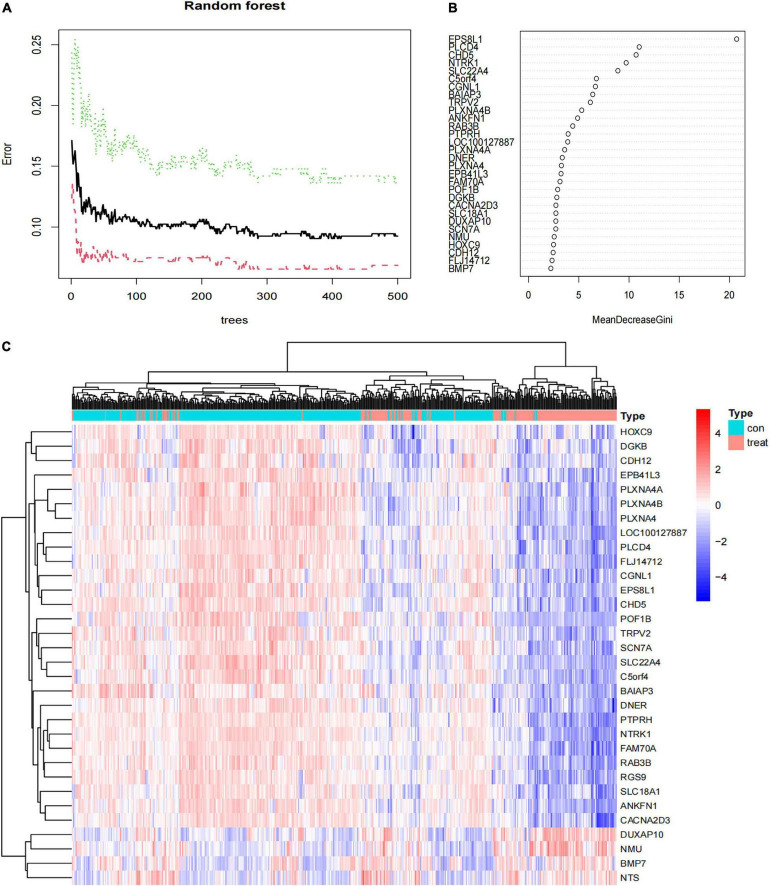
The results of the top 30 genes screened by random forest. **(A)** The plot of performance in log scale against epoch number; **(B)** the importance of the top 30 genes ranked by the mean decrease of accuracy; **(C)** heat map of the top 30 genes.

Next, the 94 DEGs wad input into the RF classifier. The relationship plot between the number of decision trees and the model error is shown in Figure 5A; 500 trees were selected as the parameter of the model. Finally, we chose 290 trees which showed a minimum error in the model. And then, 32 DEGs with an importance greater than 2 were identified as the candidate genes for further analysis. Among the 32 variables, EPS8L1, PLCD4, CHD5, NTRK1, and SLC22A4 were the most important (Figure 5B). The k-means unsupervised clustering was performed in the training cohort based on these 32 important variables. Figure 5C displays that the 32 feature genes could be used to distinguish between the low- and high-risk category samples in 498 samples.

### Construction and Validation of Artificial Neural Network Model

**FIGURE 6:**
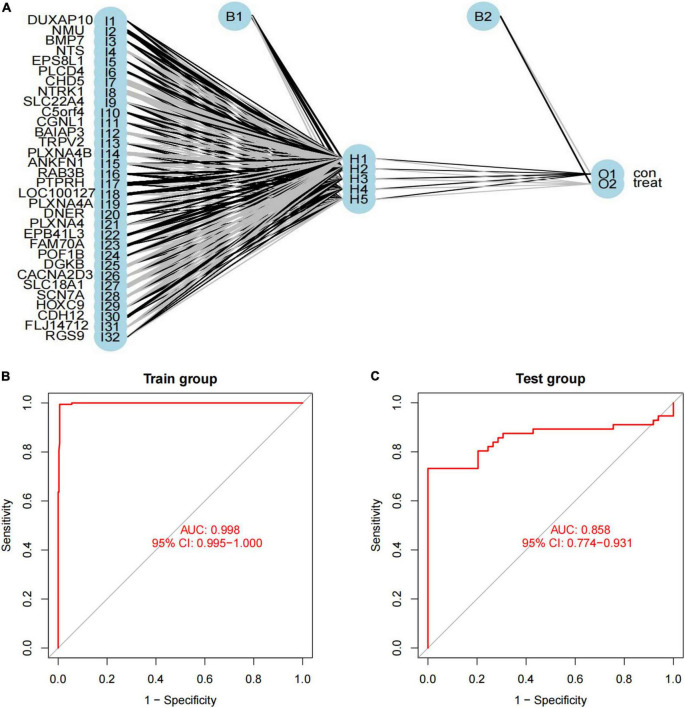
Construction of artificial neural network (ANN) model. **(A)** ANN model has 30 inputs, 5 hidden neurons, and 2 outputs. In this case, the 30 inputs represent the category values of 30 feature DEGs; **(B,C)** the receiver operating characteristic curve of the predictive model in **(B)** training dataset and **(C)** validation dataset.

We used a training cohort to construct an ANN model based on the ANN algorithm by using “neuralnet” R package (Figure 6A). First, the expression data of feature genes were normalized. And then, the weight value of each feature gene was converted into Gene Score. Supplementary Table 2 shows the Gene Weight of each feature gene. One hidden layer and five neurons were selected in the model. Using the “ROCR” R package, the model classification performance was displayed by the receiver operating characteristic (ROC) curve (Figure 6B). The areas under the ROC curves (AUC) of our model were close to 1 (AUC: 0.998 95% CI: 0.995–1.000), confirming the robustness of the model.

### Verification of Artificial Neural Network Model

**FIGURE 7:**
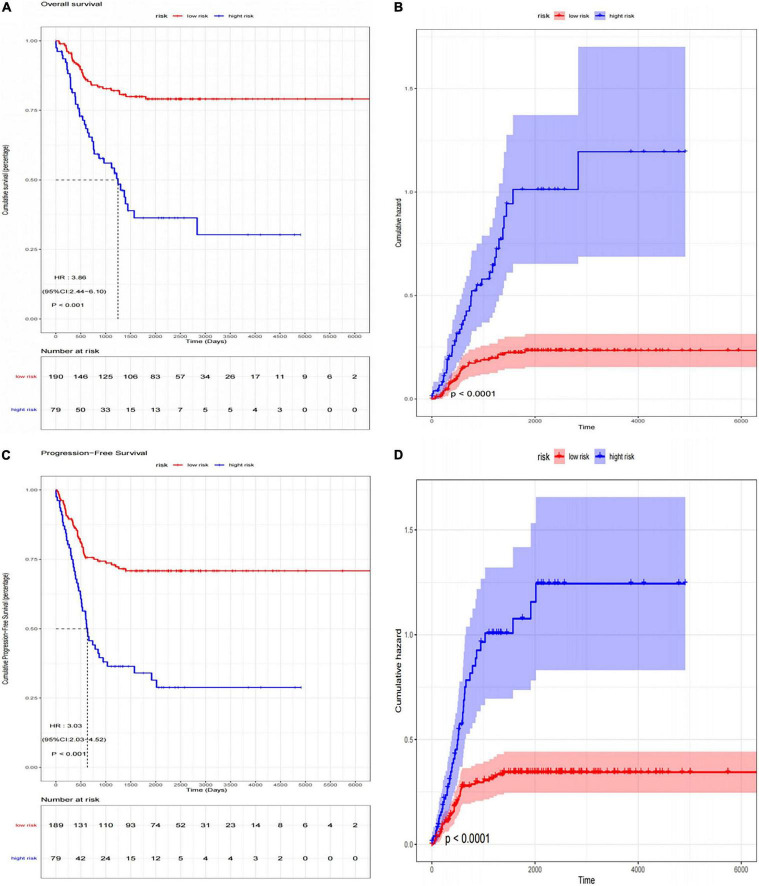
The Kaplan–Meier survival analysis for neuroblastoma patients divided into high-risk subgroup and low-risk subgroup. **(A)** Kaplan–Meier survival curves (log-rank test, HR: 3.86 (95% CI: 2.44–6.10), *p* < 0.001) and **(B)** cumulative risk curves (log-rank test, *p* < 0.001) of OS for high-risk and low-risk subgroup in validation set; **(C)** Kaplan–Meier survival curves [log-rank test, HR: 3.03 (95% CI: 2.03–4.52), *p* < 0.001] and **(D)** cumulative risk curves (log-rank test, *p* < 0.001) of PFS for high-risk and low-risk subgroup in validation set.

We used another dataset as the testing cohort to verify the classification efficiency of the model score. In the same way as the training cohort (GSE73517), the gene score of the testing cohort was calculated. The AUC was 0.858 (95% CI: 0.774-0.931), which indicated the stability and validity of the ANN model (Figure 6C). Subsequently, we divided the validation set into two groups according to the ANN model, and evaluated the disease progression and overall survival of the two groups. Kaplan–Meier plotter was used to analyze the subgroups, named low-risk subgroup and high-risk subgroup, and the results showed that the overall survival of patients in the low-risk subgroup was significantly better than those in the high-risk subgroup [log rank test, HR: 3.86 (95% CI: 2.44–6.10), *p* < 0.001; Figure 7A]. And there was a statistically significant difference in the cumulative risk of OS between the two subgroups (log-rank test, *p* < 0.001; Figure 7B). Additionally, the progression-free survival was further assessed in the validation set. The results showed that the high-risk subgroup had significant short progression-free survival time [log rank test, HR: 3.03 (95% CI: 2.03–4.52), *p* < 0.001; Figure 7C]. And there was a statistically significant difference in the cumulative risk of PFS between the two subgroups (log-rank test, *p* < 0.001; Figure 7D).

**FIGURE 8:**
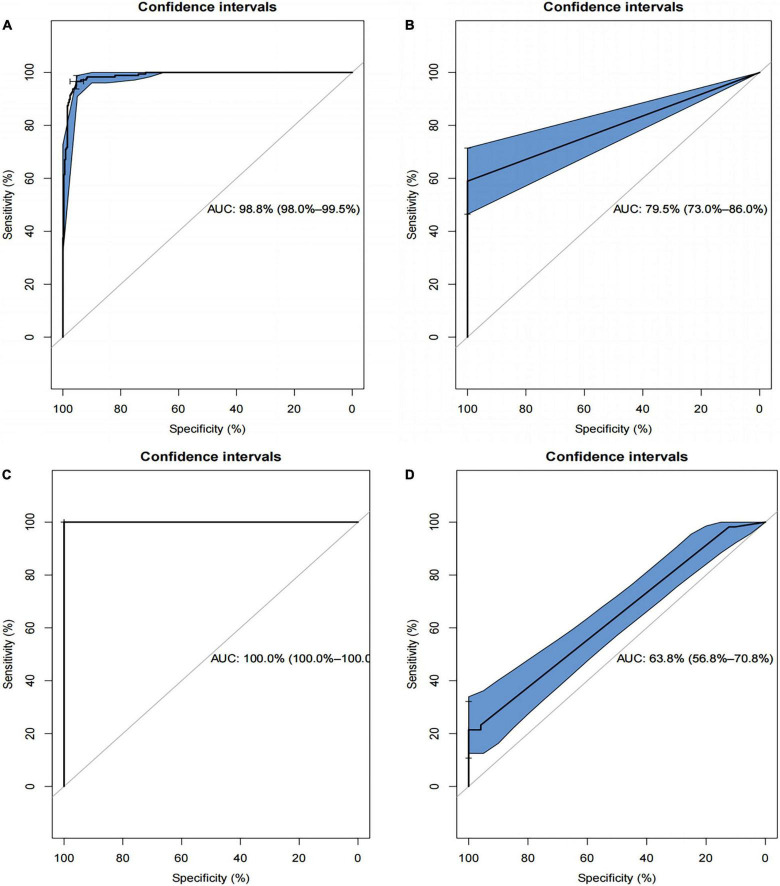
Receiver operating characteristics (ROCs) curve for the SVM-based and the XGBoost-based model in the training and test datasets. **(A)** The ROC curve for SVM-based model in training set; **(B)** the ROC curve for SVM-based model in test set; **(C)** the ROC curve for XGBoost-based model in the training set; **(D)** the ROC curve for XGBoost-based model in the test set.

The ROC plots of the SVM-based and the XGBoost-based models in the training and test datasets are shown in Figures 8A–D, respectively. Using bootstrapping validation, the area under the ROC curve values for the SVM model were found to be 0.988 (95% CI: 0.980–0.995) and 0.795 (95% CI: 0.730–0.860) in the train and test groups, respectively. The area under the ROC curve values for the XGBoost model were found to be 1 (95% CI: 1–1) and 0.638 (95% CI: 0.568–0.708) in the train and test groups, respectively. This indicates the two models performed well in the train groups, but not in the test groups.

## Discussion

Early and accurate differentiation of neuroblastoma patients between high-risk and non-high-risk groups has good clinical value, as there are significant differences in treatment and prognosis between the two groups (36). At present, the grouping is mainly based on histology and immunohistochemistry, but such diagnosis is often based on surgery and the accuracy is still insufficient (37). In addition, the changes in cancer first appear at the gene level, and histological changes are always a dynamic process, so the results are prone to bias. In recent years, the development of machine learning algorithms and the explosion of gene expression data in public databases have provided new biomarker approaches to disease diagnosis or prognosis. In this study, we established a disease grouping model based on high-risk grouping characteristic genes using random forest combined with the artificial neural network, providing a complementary tool for elucidating the biological process of high-risk neuroblastoma and risk stratification of cancer. Our goal is to establish a prediction model that can accurately assess the risk of patients before treatment, accurately predict the prognosis of patients, help us develop a more appropriate self-management program, and rationally allocate medical resources.

First, we identified 88 down-regulated DEGs and 6 up-regulated DEGs in the dataset GSE49710 between the high-risk and non-high-risk samples. GO and KEGG enrichment analysis revealed that high-risk neuroblastoma-associated DEGs were involved in multiple GO terms and pathways, reflecting the dynamics and complexity of their pathogenesis, modulation of chemical synaptic transmission, regulation of transsynaptic signaling, and neurotransmitter transport/uptake/reuptake. The modulation of chemical synaptic transmission, regulation of transsynaptic signaling, and neurotransmitter transport/uptake/reuptake are important in the function of the nervous system. We assessed differences in immune cells in the tumor microenvironment between high-risk and non-high-risk groups and found a higher proportion of Plasma cells, memory B cells, activated memory CD4 T cells, Neutrophils and a lower proportion of resting memory CD4 T cells, M2 macrophages, activated mast cells were generally contained in high-risk category primary neuroblastomas tissues, suggesting that this is an immune apathetic tumor (38). We then identified 32 characteristic genes for high-risk neuroblastoma based on random forest algorithm, among which CHD5 is a tumor suppressor at 1P36, which is often lost or silenced in poor prognostic neuroblastoma (NB) and many adult cancers (39). A recent study has confirmed that CHD5 is a metastasis suppressor in NB. It is well known that amplification of myC-N proto-oncogene (MYCN) is a major driver of NB aggressiveness and that high expression of neurotrophic factor receptor NTRK1/TrkA is associated with mild disease course (40). However, the roles of most of the signature genes in neuroblastoma are still unclear and require further study. Next, we used the artificial neural network algorithm to calculate the weight value of 32 features, and calculate the gene score of each tumor through the weight value of each feature gene of each patient, to distinguish tumors in high-risk and non-high-risk groups samples.

The biggest highlight of this study is the innovative combination of random forest and artificial neural network, which improves the predictive ability of the high-risk neuroblastoma prediction model, and creatively achieves good results in terms of predictive ability. The prediction ANN model based on gene expression data in this study showed high overall accuracy and precision in both the training set and the test set (AUC = 0.998 in GSE49710 and AUC = 0.858 in GSE73517). Moreover, the SVM-based and the XGBoost-based model performed well in the train groups, but not in the test groups (AUC = 0.795 and 0.638, respectively). This indicated that the classification accuracy of the ANN-based model had a better predictive ability and generalization ability. In addition, the ANN-based model divided the validation set into high-risk subgroup and low-risk subgroup, and survival analysis results show that OS and PFS of high-risk subgroup were significantly worse, and cumulative risks were significantly higher. This proved that our model can predict the prognosis of patients well. Machine learning is more reliable and accurate in data analysis. The collection of gene expression profiles of neuroblastoma is easier than clinical patient information, more objective, and more cost-effective. The AUC of the independent validation set prediction model reached 0.858, which also confirmed the universal applicability of the scoring system we established. The screening results of the RF classifier showed that EPS8L1, PLCD4, CHD5, NTRK1, and SLC22A4 were the most characteristic DEG genes among high-risk neuroblastoma-related genes (39, 41–44). It is worth noting that the role of these genes in the occurrence and development of neuroblastoma is still unclear, and more basic studies are needed in the future to clarify the mechanism of these genes in neuroblastoma, which will help us to have a deeper and more accurate understanding of the disease and may find therapeutic targets for the disease. Importantly, future work will focus on the application of a disease risk grouping scoring system based on feature genes in neuroblastoma.

However, there are still some limitations to our study. First of all, our training set and verification set are small sample data. Due to the limited sample size, we did not perform 10-fold cross-validation in the neural network analysis. In addition, these data are from retrospective studies. Nevertheless, our model has good classification performance, and more convincing data sets and machine learning algorithms will be needed to build diagnostic models for individual organization types in the future. Furthermore, we used microarray data but not RNA sequencing (RNA-seq) for validation, but we did not find RNA-seq data available for analysis. As RNA-seq is more likely to find novel genes, it should be included in our future work. Last but not the least, we have evaluated the performance of the ANN model by comparing the predictive results of the XGBoost model and SVM model. All the three classifiers employed in the study are state-of-the-art machine learning techniques that show good performances in various applications. However, some recent machine learning or feature selection methods are not discussed in our study, for example, Huang et al. proposed a novel method for gene selection and phenotype classification and an efficient tool for survival analysis and biomarker selection (8, 9).

## Data Availability Statement

The datasets presented in this study can be found in online repositories. The names of the repository/repositories and accession number(s) can be found below: https://www.ncbi.nlm.nih.gov/, GSE49710, GSE73517, GSE85047.

## Author Contributions

JS and SY conceived and designed the study. SY and LZ acquired and analyzed the validation set, reviewed and rewrote the manuscript. SY and HL acquired and analyzed the high throughput data, and contributed analysis tools. SY and XJ wrote the manuscript. All authors read and approved the final manuscript.

## Conflict of Interest

The authors declare that the research was conducted in the absence of any commercial or financial relationships that could be construed as a potential conflict of interest.

## Publisher’s Note

All claims expressed in this article are solely those of the authors and do not necessarily represent those of their affiliated organizations, or those of the publisher, the editors and the reviewers. Any product that may be evaluated in this article, or claim that may be made by its manufacturer, is not guaranteed or endorsed by the publisher.

## Footnotes

1
https://www.ncbi.nlm.nih.gov/geo/

2
https://metascape.org/

3
https://string-db.org/
