# Supplementary material for: Feature Genes in Neuroblastoma Distinguishing High-Risk and Non-high-Risk Neuroblastoma Patients: Development and Validation Combining Random Forest With Artificial Neural Network
Source: Front Med (Lausanne). 2022 Jul 15;9:882348. doi: 10.3389/fmed.2022.882348 (PMC9336509; doi:10.3389/fmed.2022.882348)
Supplement: Supplementary file 1 [file Data_Sheet_1.doc]

Diff genes

library(limma)

library(pheatmap)

inputFile="TCGA.TPM.txt"

logFCfilter=2

adj.P.Val.Filter=0.05

setwd("C:\\ANN")

rt=read.table(inputFile, header=T, sep="\t", check.names=F)

rt=as.matrix(rt)

rownames(rt)=rt[,1]

exp=rt[,2:ncol(rt)]

dimnames=list(rownames(exp),colnames(exp))

data=matrix(as.numeric(as.matrix(exp)),nrow=nrow(exp),dimnames=dimnames)

data=avereps(data)

data=data[rowMeans(data)>0,]

sampleName1=c()

files=dir()

files=grep("s1.txt$", files, value=T)

for(file in files){

rt=read.table(file, header=F, sep="\t", check.names=F)

geneNames=as.vector(rt[,1])

uniqGene=unique(geneNames)

sampleName1=c(sampleName1, uniqGene)

}

sampleName2=c()

files=dir()

files=grep("s2.txt$", files, value=T)

for(file in files){

rt=read.table(file, header=F, sep="\t", check.names=F)

geneNames=as.vector(rt[,1])

uniqGene=unique(geneNames)

sampleName2=c(sampleName2, uniqGene)

}

conData=data[,sampleName1]

treatData=data[,sampleName2]

data=cbind(conData,treatData)

conNum=ncol(conData)

treatNum=ncol(treatData)

Type=c(rep("con",conNum),rep("treat",1))

design <- model.matrix(~0+factor(Type))

colnames(design) <- c("con","treat")

fit <- lmFit(data,design)

cont.matrix<-makeContrasts(treat-con,levels=design)

fit2 <- contrasts.fit(fit, cont.matrix)

fit2 <- eBayes(fit2)

allDiff=topTable(fit2,adjust='fdr',number=200000)

allDiffOut=rbind(id=colnames(allDiff),allDiff)

write.table(allDiffOut, file="all.txt", sep="\t", quote=F, col.names=F)

outData=rbind(id=paste0(colnames(data),"_",Type),data)

write.table(outData, file="normalize.txt", sep="\t", quote=F, col.names=F)

diffSig=allDiff[with(allDiff, (abs(logFC)>logFCfilter & adj.P.Val < adj.P.Val.Filter )), ]

diffSigOut=rbind(id=colnames(diffSig),diffSig)

write.table(diffSigOut, file="diff.txt", sep="\t", quote=F, col.names=F)

diffGeneExp=data[row.names(diffSig),]

diffGeneExpOut=rbind(id=paste0(colnames(diffGeneExp),"_",Type), diffGeneExp)

write.table(diffGeneExpOut, file="diffGeneExp.txt", sep="\t", quote=F, col.names=F)

#heatmap

geneNum=50

diffSig=diffSig[order(as.numeric(as.vector(diffSig$logFC))),]

diffGeneName=as.vector(rownames(diffSig))

diffLength=length(diffGeneName)

hmGene=c()

if(diffLength>(2*geneNum)){

hmGene=diffGeneName[c(1:geneNum,(diffLength-geneNum+1):diffLength)]

}else{

hmGene=diffGeneName

}

hmExp=data[hmGene,]

Type=c(rep("Con",conNum),rep("Treat",treatNum))

names(Type)=colnames(data)

Type=as.data.frame(Type)

pdf(file="heatmap.pdf", width=10, height=8)

pheatmap(hmExp,

annotation=Type,

color = colorRampPalette(c("blue", "white", "red"))(50),

cluster_cols =F,

show_colnames = F,

scale="row",

fontsize = 8,

fontsize_row=7,

fontsize_col=8)

dev.off()

#volcano

library(ggplot2)

logFCfilter=2

adj.P.Val.Filter=0.05

inputFile="all.txt"

setwd("C:\\ANN")

rt=read.table(inputFile, header=T, sep="\t", check.names=F)

Sig=ifelse((rt$adj.P.Val<adj.P.Val.Filter) & (abs(rt$logFC)>logFCfilter), ifelse(rt$logFC>logFCfilter,"Up","Down"), "Not")

rt=cbind(rt, Sig=Sig)

p=ggplot(rt, aes(logFC, -log10(adj.P.Val)))+

geom_point(aes(col=Sig))+

scale_color_manual(values=c("green", "black", "red"))+

xlim(-5,5)+

labs(title = " ")+

geom_vline(xintercept=c(-logFCfilter,logFCfilter), col="blue", cex=1, linetype=2)+

geom_hline(yintercept= -log10(adj.P.Val.Filter), col="blue", cex=1, linetype=2)+

theme(plot.title=element_text(size=16, hjust=0.5, face="bold"))

p=p+theme_bw()

pdf(file="volcano.pdf", width=6, height=5.1)

print(p)

dev.off()

#GO

library("clusterProfiler")

library("org.Hs.eg.db")

library("enrichplot")

library("ggplot2")

library(GOplot)

pvalueFilter=0.05

qvalueFilter=0.05

colorSel="qvalue"

if(qvalueFilter>0.05){

colorSel="pvalue"

}

setwd("C:\\ANN")

rt=read.table("diff.txt", header=T, sep="\t", check.names=F)

colnames(rt)[1]="Gene"

genes=as.vector(rt[,1])

entrezIDs=mget(genes, org.Hs.egSYMBOL2EG, ifnotfound=NA)

entrezIDs=as.character(entrezIDs)

gene=entrezIDs[entrezIDs!="NA"]

kk=enrichGO(gene=gene,OrgDb=org.Hs.eg.db, pvalueCutoff=1, qvalueCutoff=1, ont="all", readable =T)

GO=as.data.frame(kk)

GO=GO[(GO$pvalue<pvalueFilter & GO$qvalue<qvalueFilter),]

write.table(GO,file="GO.txt",sep="\t",quote=F,row.names = F)

#KEGG

library("clusterProfiler")

library("org.Hs.eg.db")

library("enrichplot")

library("ggplot2")

library(GOplot)

pvalueFilter=0.05

qvalueFilter=0.05

colorSel="qvalue"

if(qvalueFilter>0.05){

colorSel="pvalue"

}

setwd("C:\\biowolf\\neuralDiagnostic\\10.KEGG")

rt=read.table("diff.txt", header=T, sep="\t", check.names=F)

colnames(rt)[1]="Gene"

genes=as.vector(rt[,1])

entrezIDs=mget(genes, org.Hs.egSYMBOL2EG, ifnotfound=NA)

entrezIDs=as.character(entrezIDs)

rt=cbind(rt,entrezID=entrezIDs)

gene=entrezIDs[entrezIDs!="NA"]

kk <- enrichKEGG(gene=gene, organism="hsa", pvalueCutoff=1, qvalueCutoff=1)

KEGG=as.data.frame(kk)

KEGG$geneID=as.character(sapply(KEGG$geneID,function(x)paste(rt$Gene[match(strsplit(x,"/")[[1]],as.character(rt$entrezID))],collapse="/")))

KEGG=KEGG[(KEGG$pvalue<pvalueFilter & KEGG$qvalue<qvalueFilter),]

write.table(KEGG, file="KEGG.txt", sep="\t", quote=F, row.names = F)

#RandomForest

library(randomForest)

set.seed(123456)

inputFile="diffGeneExp.txt"

setwd("C:\\ANN")

data=read.table(inputFile, header=T, sep="\t", check.names=F, row.names=1)

data=t(data)

group=gsub("(.*)\\_(.*)", "\\2", row.names(data))

rf=randomForest(as.factor(group)~., data=data, ntree=500)

pdf(file="forest.pdf", width=6, height=6)

plot(rf, main="Random forest", lwd=2)

dev.off()

optionTrees=which.min(rf$err.rate[,1])

optionTrees

rf2=randomForest(as.factor(group)~., data=data, ntree=optionTrees)

importance=importance(x=rf2)

pdf(file="geneImportance.pdf", width=6.2, height=5.8)

varImpPlot(rf2, main="")

dev.off()

rfGenes=importance[order(importance[,"MeanDecreaseGini"], decreasing = TRUE),]

rfGenes=names(rfGenes[rfGenes>2])

write.table(rfGenes, file="rfGenes.txt", sep="\t", quote=F, col.names=F, row.names=F)

sigExp=t(data[,rfGenes])

sigExpOut=rbind(ID=colnames(sigExp),sigExp)

write.table(sigExpOut, file="rfGeneExp.txt", sep="\t", quote=F, col.names=F)

#Gene score

library(limma)

expFile="rfGeneExp.txt"

diffFile="diff.txt"

setwd("C:\\ANN")

rt=read.table(expFile, header=T, sep="\t", check.names=F)

rt=as.matrix(rt)

rownames(rt)=rt[,1]

exp=rt[,2:ncol(rt)]

dimnames=list(rownames(exp),colnames(exp))

data=matrix(as.numeric(as.matrix(exp)),nrow=nrow(exp),dimnames=dimnames)

data=avereps(data)

diffRT=read.table(diffFile, header=T, sep="\t", check.names=F, row.names=1)

diffRT=diffRT[row.names(data),]

dataUp=data[diffRT[,"logFC"]>0,]

dataDown=data[diffRT[,"logFC"]<0,]

dataUp2=t(apply(dataUp,1,function(x)ifelse(x>median(x),1,0)))

dataDown2=t(apply(dataDown,1,function(x)ifelse(x>median(x),0,1)))

outTab=rbind(dataUp2, dataDown2)

outTab=rbind(id=colnames(outTab), outTab)

write.table(outTab, file="geneScore.txt", sep="\t", quote=F, col.names=F)

#ANN MODEL

library(neuralnet)

library(NeuralNetTools)

set.seed(12345678)

inputFile="geneScore.txt"

setwd("C:\\ANN")

data=read.table(inputFile, header=T, sep="\t", check.names=F, row.names=1)

data=as.data.frame(t(data))

group=gsub("(.*)\\_(.*)", "\\2", row.names(data))

data$con=ifelse(group=="con", 1, 0)

data$treat=ifelse(group=="treat", 1, 0)

fit=neuralnet(con+treat~., data, hidden=5)

fit$result.matrix

fit$weight

#plot(fit)

pdf(file="neuralnet.pdf", width=9, height=7)

plotnet(fit)

dev.off()

net.predict=compute(fit, data)$net.result

net.prediction=c("con", "treat")[apply(net.predict, 1, which.max)]

predict.table=table(group, net.prediction)

predict.table

conAccuracy=predict.table[1,1]/(predict.table[1,1]+predict.table[1,2])

treatAccuracy=predict.table[2,2]/(predict.table[2,1]+predict.table[2,2])

paste0("Con accuracy: ", sprintf("%.3f", conAccuracy))

paste0("Treat accuracy: ", sprintf("%.3f", treatAccuracy))

colnames(net.predict)=c("con", "treat")

outTab=rbind(id=colnames(net.predict), net.predict)

write.table(outTab, file="neural.predict.txt", sep="\t", quote=F, col.names=F)

#ROC

library(pROC)

inputFile="neural.predict.txt"

setwd("C:\\ANN")

rt=read.table(inputFile, header=T, sep="\t", check.names=F, row.names=1)

y=gsub("(.*)\\_(.*)", "\\2", row.names(rt))

y=ifelse(y=="con", 0, 1)

roc1=roc(y, as.numeric(rt[,2]))

ci1=ci.auc(roc1, method="bootstrap")

ciVec=as.numeric(ci1)

pdf(file="ROC.pdf", width=5, height=5)

plot(roc1, print.auc=TRUE, col="red", legacy.axes=T, main="Train group")

text(0.39, 0.43, paste0("95% CI: ",sprintf("%.03f",ciVec[1]),"-",sprintf("%.03f",ciVec[3])), col="red")

dev.off()
